# Deep learning prediction of overall survival in glioblastoma patients using pre-chemoradiotherapy MRI and clinical data

**DOI:** 10.3389/fradi.2026.1963539

**Published:** 2026-10-13

**Authors:** Noémie N. Moreau, Alexis Desmonts, Cyril Jaudet, Thomas Leleu, Alexandre G. Leclercq, Carole Brunaud, Dinu Stefan, Samuel Valable, Alexis Lechervy, Aurélien Corroyer-Dulmont

**Affiliations:** 1 Artificial Intelligence Department, Centre François Baclesse, Caen, France; 2 Université de Caen Normandie, CNRS, Normandie Univ, ISTCT UMR6030, CYCERON, Caen, France; 3 Medical Physics Department, Centre François Baclesse, Caen, France; 4 Radiation Oncology Department, Centre François Baclesse, Caen, France; 5 UMR GREYC, Normandie Univ, UNICAEN, ENSICAEN, CNRS, Caen, France

**Keywords:** deep learning, glioblastoma, MRI, radiotherapy, survival outcome

## Abstract

**Purpose:**

Glioblastoma (GBM) exhibits variable treatment response and patients shown a median survival of 15 months. A major challenge lies in the difficulty of reliably assessing treatment response at an early stage, as it can usually only be evaluated on anatomical MRI performed up to three months after the start of treatment, when early interpretation may be complicated by the risk of pseudoprogression. Accurately predicting short-term and long-term survivors before radiotherapy and chemotherapy is therefore crucial. The objective of this study is to develop an AI model designed to predict survival outcomes based exclusively on pre-chemoradiotherapy MRI and clinical data.

**Material and methods:**

A total of 1,575 retrospective T1-Gd brain images were obtained from 175 patients treated for GBM between 2018 and 2023. A ResNet-51Q model pretrained on ImageNet was fine-tuned on pre-chemoradiotherapy MRI using transfer learning. To incorporate clinical data, deep hybrid learning (DHL) models were trained.

**Results:**

At patient-level, the AI model achieved 81.08% (CI 95%: 67.57–91.89), 68.42% (CI 95%: 47.06–88.89) and 94.44% (CI 95%: 81.82–1.00) for accuracy, sensitivity, and specificity, respectively, for the classification of short-term and long-term survivors using only pre-chemoradiotherapy MRI. At patient-level, the three-class model using only pre-chemoradiotherapy MRI, achieved 87.18% (CI 95%: 76.92– 97.44), 87.18% (CI 95%: 76.68–96.97), and 93.59% (CI 95%: 88.03–98.41) for accuracy, sensitivity, and specificity, respectively. In comparison at slice-level, the three-class model using only pre-chemoradiotherapy MRI, achieved 81.77% (CI 95%: 70.66–91.45), 81.77% (CI 95%: 71.07–91.33), and 90.88% (CI 95%: 85.25–95.74) for accuracy, sensitivity, and specificity, respectively. In comparison the DHL model with clinical data achieved an accuracy of 84.62% (CI 95%: 71.79–94.87), a sensitivity of 84.62% (CI 95%: 72.54–95.24) and a specificity of 92.31% (CI 95%: 86.07–97.53) and 76.32% (CI 95%: 63.16–89.47), 76.11% (CI 95%: 61.63–88.75) and 92.06% (CI 95%: 87.51–96.23) for four overall survival classification. For multiclass tasks, metrics were calculated using a macro-averaged approach.

**Conclusion:**

The combination of pre-chemoradiotherapy MRI and clinical data using the DHL model for early survival prediction; however, adding clinical variables yielded comparable prognostic performance rather than a demonstrable enhancement over imaging features alone. The use of AI models to predict survival outcome in GBM from pre-chemoradiotherapy data may support pre-chemoradiotherapy prognostic stratification.

## Introduction

Glioblastoma (GBM) is the most frequent primary malignant brain tumor in adults. Although the standard treatment consists of maximal surgical resection followed by radiotherapy and concomitant chemotherapy with temozolomide (the Stupp protocol) (1), prognosis remains poor. The median overall survival remains approximately 12–15 months (2). However, despite these therapeutic approaches, considerable inter-patient heterogeneity in terms of treatment efficacy and overall survival remains. Some patients have a short overall survival, while a minority survive beyond five years (3).

Magnetic resonance imaging (MRI) plays a key role in diagnosis and also in evaluating treatment response by measuring post-treatment lesion size according to the Response Assessment in Neuro-Oncology (RANO) criteria (4, 5). However, these criteria have an important limitation in the early post-radiotherapy period. Size-based assessment cannot reliably distinguish true progression from pseudoprogression and suspected progression must be confirmed by a follow-up MRI (typically 4–8 weeks later) or by histopathological evidence, delaying the definitive characterization of tumor progression. In this clinical context, there is a critical need for early survival classification from pre-chemoradiotherapy MRI, which could identify patients at high risk of poor outcome before therapy begins. By stratifying patients into short-term and long-term survivors based on their pre-chemoradiotherapy MRI, prognosis can be anticipated before treatment initiation, enabling timely adjustments within a personalized medicine framework in the future (6, 7).

Several studies have investigated survival prediction in brain tumor patients. Similar methods were developed for glioblastomas (8, 9). One approach is to predict treatment efficacy and survival outcome using imaging biomarkers. Advanced molecular imaging can detect treatment-induced metabolic changes far earlier than anatomical changes become visible. In recurrent glioblastoma treated with anti-angiogenic therapy, MR spectroscopy metabolite changes measured as early as one day after treatment initiation can provide better insight into how therapy affects the tumor microenvironment and help predict patient outcomes (10). Another study has shown that 18F-FDG PET can capture early changes reflecting whether a drug engages its target within days of administration (11). In newly diagnosed glioblastoma, metabolic changes measured by MR spectroscopy during chemoradiotherapy have been shown to be associated with survival (12). These results suggest that early imaging changes may enable prediction of treatment response before size-based assessment with the RANO criteria. Radiomics and artificial intelligence (AI) have been investigated to address the challenge of predicting survival outcome. One recent study aimed to predict survival of glioblastoma at eight months after radiotherapy (13). However, the AI model used post-treatment MRI, which limits personalization as treatment has already been administered. Some studies indicate that combining imaging data with clinical information improves model performance. Nevertheless, these studies often use small cohorts and mainly use public datasets that may not represent real clinical practice (14). Consequently, the robustness, generalizability and clinical applicability of the developed models remain constrained. It is therefore crucial to develop robust predictive tools capable of assessing survival outcome prior to treatment initiation (15). These tools should overcome the limitations of current evaluations based on anatomical MRI, facilitating more effective patient management and minimizing the risks associated with ineffective therapies. To our knowledge, no AI solution is currently integrated into routine practice to predict survival outcome prior to treatment.

This study aimed to develop deep learning model using pre-chemoradiotherapy MRI, to predict survival outcomes at various time points after diagnosis. Firstly, a binary model predicting short versus long survivor was built and then, models predicting three to four classes were implemented.

## Materials and methods

### Ethics

This retrospective study received approval from the Centre François Baclesse institutional review board (internal ethics committee “CSE” data department, health data hub study number: F20240111162856). The study was conducted in compliance with the principles of the Declaration of Helsinki and the MR-004 guidelines established by the French National Institute for Health Data (INDS) for health research. Informed consent was obtained from all patients for the use of their data.

### Patient cohort

The initial cohort comprised 385 patients (age ≥ 18 years old) diagnosed with glioblastoma according to the 2016 and 2021 WHO classifications of central nervous system tumors (16, 17) between January 2018 and December 2023. It is important to note that one patient was classified as IDH-mutant GBM under WHO 2016. Under WHO 2021, this entity is instead considered an astrocytoma. This proportion represents 0.57% of the entire cohort. We excluded 35 patients enrolled in a clinical trial, 56 patients with several treatment of radiotherapy, 72 patients with missing pre-chemoradiotherapy MRI, or with an MRI not acquired at our oncology center, 42 patients who were still alive or lost to follow-up, and 5 with missing clinical data. After these exclusions, 175 patients were included in the final cohort (Supplementary Figure S1).

In this study, overall survival prediction is treated as a classification task. To train the model, we only included deceased patients, because their exact survival times are unknown, right-censored patients cannot be assigned the deterministic ground-truth labels required by our multiclass classification framework, and were therefore excluded from training. To achieve class balance, survival thresholds were derived exclusively from the training cohort and subsequently applied to the validation and test cohorts. Short-term and long-term survivors were categorized according to the median survival of the training cohort (Supplementary Figure S2a). The median cohort survival was 332 days, and patients with shorter or longer survival were stratified accordingly. Details of the study population for binary classes are presented in Table 1.

**Table 1:** Patient cohort description for all patients and for short-term or long-term survivors defined as < or ≥332 days, respectively.

Category	All patients (*n* = 175)	Short-term (*n* = 86)	Long-term (*n* = 89)
Survival (days)	mean: 363.4 median: 332.0 (IQR: 349.0) min-max: [42.0–1600.0]	mean: 163.6 median: 137.0 (IQR: 119.8) min-max: [42.0–329.0]	mean: 556.5 median: 492.0 (IQR: 264.0) min-max: [332.0–1600.0]
Sex, *n* (%)	Female: 74 (42.3%) Male: 101 (57.7%)	Female: 39 (45.3%) Male: 47 (54.7%)	Female: 35 (39.3%) Male: 54 (60.7%)
Age at diagnosis (years)	mean: 67.7 median: 332.0 (IQR: 349.0) min-max: [25.5–88.9]	mean: 70.4 median: 71.3 (IQR: 10.2) min-max: [25.5–88.9]	mean: 65.1 median: 65.6 (IQR: 13.6) min-max: [42.3–83.7]
Resection type, *n* (%)	Biopsy: 69 (39.4%) Subtotal: 62 (35.4%) Gross-total: 44 (25.1%)	Biopsy: 51 (59.3%) Subtotal: 28 (32.6%) Gross-total: 7 (8.1%)	Biopsy: 18 (20.2%) Subtotal: 34 (38.2%) Gross-total: 37 (41.6%)
WHO performance status, *n* (%)	0: 27 (15.4%) 1: 95 (54.3%) 2: 45 (25.7%) 3: 8 (4.6%) 4: 0 (0.0%) 5: 0 (0.0%)	0: 7 (8.1%) 1: 40 (46.5%) 2: 32 (37.2%) 3: 7 (8.1%) 4: 0 (0.0%) 5: 0 (0.0%)	0: 20 (22.5%) 1: 55 (61.8%) 2: 13 (14.6%) 3: 1 (1.1%) 4: 0 (0.0%) 5: 0 (0.0%)

To challenge the potential of the AI model, three and four classes of overall survival were also defined to ensure balanced classes using the tercile (Supplementary Figure S2b) and quartile thresholds of the training cohort (Supplementary Figure S2c), the corresponding patient cohorts are described in Supplementary Tables S1, S2.

### Magnetic resonance imaging (MRI) acquisition

MRI was performed on AREA/VIDA SIEMENS 1.5/3 Tesla magnets using standard post-Gadolinium (Gd) T1 brain sequences, as described previously (18). A total of 36,400 2D MR images were acquired from the 175 patients. For this study, we targeted the central region of the tumor, corresponding to the area of maximum diameter based on the tumor delineation (GTV). Nine central slices were selected from this zone (representing approximately 20% of the tumor based on the GTV), allowing for a reproducible evaluation of the lesion. Intensity normalization was applied to account for the arbitrary intensity scale of MRI. For each patient, all slices were normalized by dividing voxel intensities by the maximum intensity value computed across all of that patient's slices.

### Artificial intelligence algorithms

#### Model development

Our deep learning (DL) model consisted of a fine-tuned ResNet-51Q (19) model, described in Supplementary Material and Methods 1. This model was developed from 1,575 unique T1-Gd brain images obtained from 175 patients. For each classification task (2, 3 and 4 classes), the images were split at the patient level into training, validation and test sets, corresponding to approximately 60%, 20% and 20% of the data respectively. For the two-class task, the training validation and test sets comprised 104 patients, 34 patients, and 37 patients. For the three-class task, they comprised 103 patients, 33 patients, and 39 patients. For the four-class task, they comprised 104 patients, 33 patients, and 38 patients. Within each task, the split was stratified by class so that every class was proportionally represented across the training, validation, and test sets, which accounts for the differing counts across tasks. In particular, the dataset was split at the patient level to avoid bias, ensuring that all slices from each patient were assigned to the same subset. The loss function used in this study was the binary cross entropy for binary model and cross entropy for multiclass models. Hyperparameters were optimized using optuna (20), which uses the Bayesian TPE (Tree-structured Parzen Estimator) approach by default. Hyperparameter optimization was performed using the validation set only. The test set was held out throughout model development and used exclusively for the final evaluation. All the code used to develop and train the models are available at: https://github.com/AurelienCD/MRI-PRED-GBM and “*Deep Learning*” folder. Because the DL model is trained on individual slices, its performance was evaluated at the slice level using the accuracy score of the test dataset, the area under the curve (AUC) of the receiver operating characteristic (ROC) curve, specificity, sensitivity, and the number of false positives. For the multiclass tasks, these metrics were computed per class using a one-vs-the-rest method. ROC curves are reported per class, and specificity, sensitivity, and AUC were macro-averaged across classes to summarize overall performance (21).

To account for the limited size of the test cohort and the non-independence of slices from the same patient, 95% confidence intervals were estimated for all metrics by patient-level bootstrap resampling with 2,000 iterations. At each iteration, patients were drawn with replacement and all their slices retained, the metric was recomputed, and confidence intervals were defined as the 2.5th and 97.5th percentiles of the bootstrap distribution. For each patient, the DL model produced a class prediction for each of the nine slices. These slice-level predictions were then aggregated into a single patient-level prediction by averaging the probability vectors, i.e., the class with the highest mean probability was assigned to the patient.

#### Machine learning

Machine learning (ML) models were used to incorporate the clinical data *i.e.,* WHO performance status, age, sex, and surgery type. The models were developed with hyperparameter optimization and cross-validation using the scikit-learn python library (21). Several ML models were tested and compared by evaluating their prediction scores: *DecisionTreeClassifier, LogisticRegression, GaussianNB, SVC, LinearDiscriminantAnalysis, XGBClassifier KNeighborsClassifier and RandomForestClassifier*. As presented in the code available at https://github.com/AurelienCD/MRI-PRED-GBM and “*Machine_Learning.ipynb*”. For each ML model, the hyperparameters were tuned to obtain a higher validation score and AUC. Feature importance was assessed using model-specific methods. We used the native “*feature_importances_”* for tree-based algorithms (*DecisionTreeClassifier*, *RandomForestClassifier*, *XGBClassifier*) and analyzed the absolute coefficients *(“coef_”)* for linear models (*LogisticRegression*, *LinearDiscriminantAnalysis*, *linear SVC*) fitted on standardized data. For algorithms lacking native metrics (*GaussianNB*, *KNeighborsClassifier*, *non-linear SVC*), we applied Permutation Importance to measure the mean decrease in predictive performance when each feature's values were randomly shuffled.

#### Deep hybrid learning

This deep hybrid learning (DHL) approach is based on a late fusion strategy (Figure 1). The final classification is driven by a hybrid input vector that integrates both high-level automated features and structured clinical data. The input for ML models is constructed as follows:

- Probabilistic outputs from DL model: For each patient, the DL model processes T1-Gd MRI slices to generate a per-slice probability distribution vector over the classes, and the vectors of the nine slices are averaged into a single patient-level vector.
- Clinical data: Structured clinical data, including WHO performance status, age, sex, and surgery type, representing patient description.

**Figure 1:**
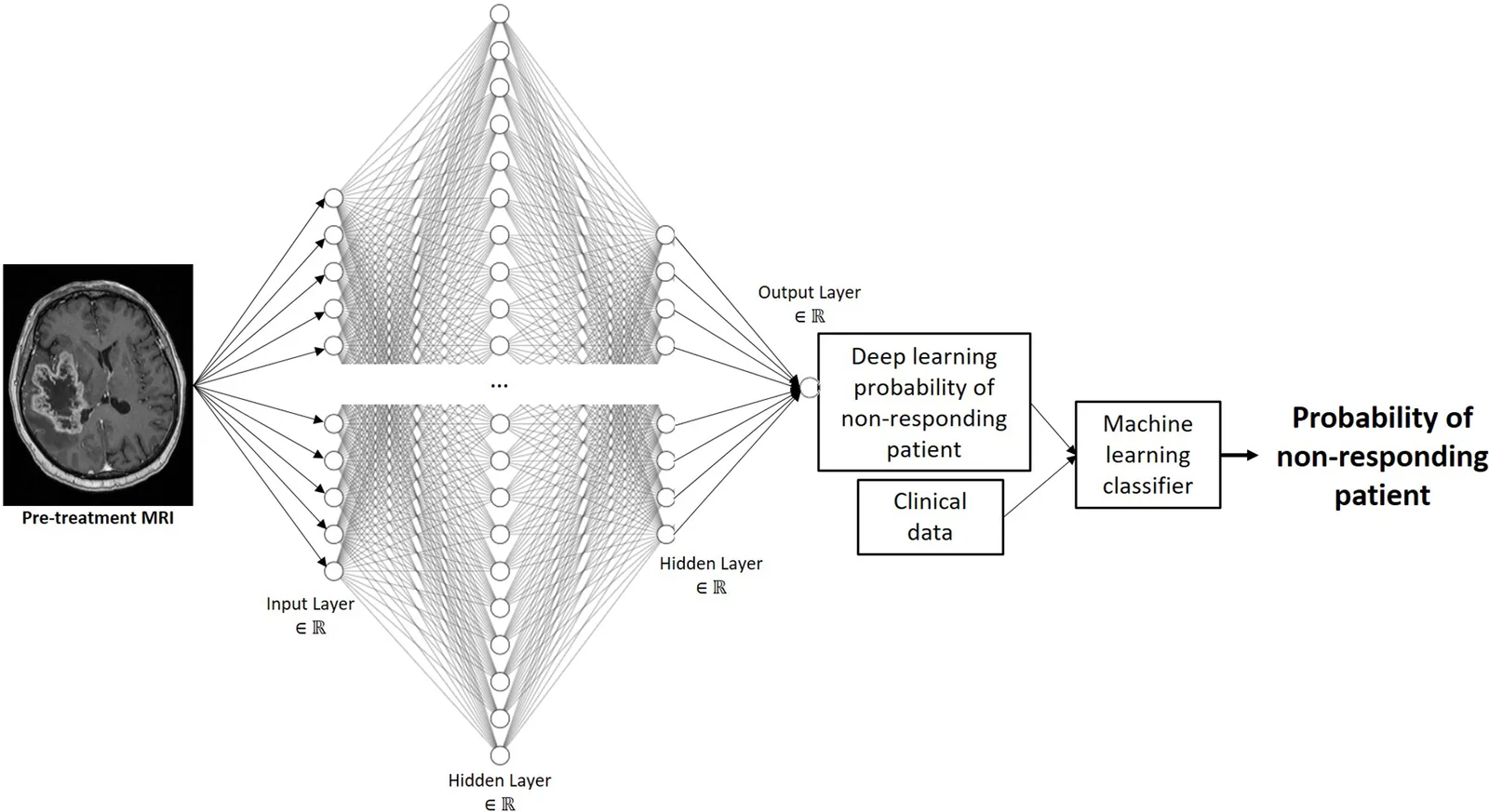
Deep hybrid learning model for glioblastoma survival outcome prediction based on pre-chemoradiotherapy MRI and clinical data (WHO performance status, age, sex and surgery type).

These two components were concatenated to form an augmented feature vector, which serves as the input for a meta-classifier (see machine learning section). This method enables us to adjust the predictions of the DL model based on the specific clinical characteristics of each patient. To avoid data leakage and prevent in-sample overconfidence bias, the DHL meta-classifier was trained using out-of-fold DL predictions generated via 5-fold cross-validation across the entire training cohort, ensuring that all nine slices from a given patient were assigned to the same fold. The same test cohort of patients was used for the final assessment of ML and DL models.

DHL model is trained at the patient level and its performance was evaluated at the patient level using the test dataset. As for DL model, the metrics were AUC of the ROC curve, specificity, sensitivity, and the number of false positives. For the multiclass tasks, these metrics were computed per class using a one-vs-the-rest method. ROC curves are reported per class, and specificity, sensitivity, and AUC were macro-averaged across classes to summarize overall performance (21).

### Statistical analyses

Analysis of variance was performed, and multiple groups comparison test was performed with ANOVA and *post-hoc* Tukey HSD test to distinguish the different quantitative metrics. Confidence intervals were computed using the Wilson score method for proportions (surgery-type-only, non-AI approach) and patient-level bootstrapping for AI models. Differences in patient-level accuracy between models were assessed using an exact McNemar test on the paired correct/incorrect classification of each patient. This test was selected because each patient constitutes an independent observation and both models were evaluated on the same patients, providing an appropriate paired comparison. To evaluate statistical differences across AUC, sensitivity and specificity, we conducted a paired patient-level bootstrap analysis. For each resample, the paired difference between models was calculated to derive empirical two-sided 95% confidence intervals and corresponding *p*-values. Differences (Δ) were considered statistically significant when the 95% confidence interval did not include zero (*p* < 0.05). All statistical analyses were performed using SciPy (22). Data visualization used Seaborn (23). All Python code used in the analysis is available at https://github.com/AurelienCD/MRI-PRED-GBM and “*Statistical_Analysis.ipynb*”.

## Results

### Prediction of short-term or long-term survivors classification

#### Based only on surgery type

Literature review and clinical routine highlight the importance of surgery completion on the efficacy of treatment and overall survival (OS) (24). For this reason, we first analyzed the ability of a non-AI based model using surgery type as input to discriminate short-term and long-term survivors. Specifically, we defined:

- True Positives (TP): Patient who underwent resection and had a survival ≥ 332 days.
- True Negatives (TN): Patient who underwent biopsy only and had a survival < 332 days.
- False Positives (FP): Patient who underwent resection but had a survival < 332 days.
- False Negatives (FN): Patient who underwent biopsy only and had a survival ≥ 332 days.

As shown in Figure 2a, surgery had a significant impact on overall survival: biopsy = 241.61 ± 191.24 days; subtotal resection = 404.32 ± 271.31 days and gross-total resection = 496.70 ± 282.83 days (*p* < 0.001 vs. biopsy group). However, although surgery impacted overall survival, the performance of a discrimination based only on that input was insufficient for use in clinical routine.

**Figure 2:**
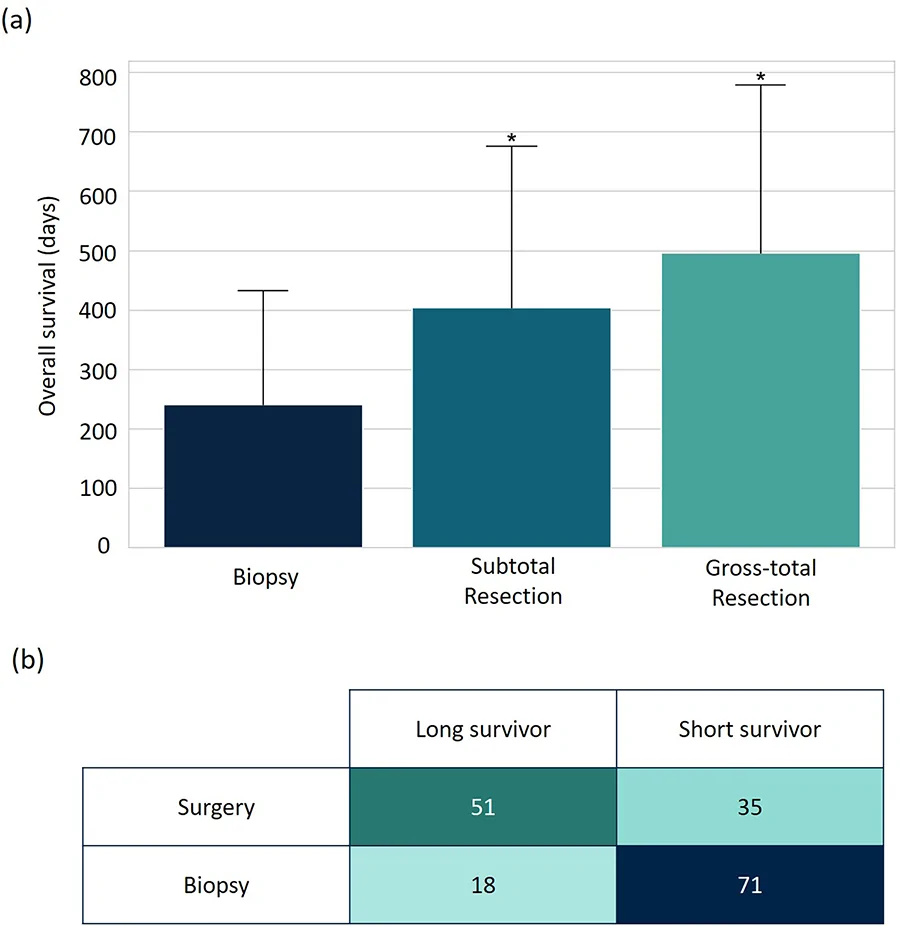
**(a)** Association between surgery type and overall survival mean ± SD. Sample sizes are n = 69, 62, 44 for biopsy, subtotal resection and gross-total resection respectively (*p < 0.001 vs. biopsy). **(b)** Confusion matrix showing the performance of surgery type alone for classifying short-and long-term survivors. SD, standard deviation.

As shown in the confusion matrix in Figure 2b, a large number of false positive (FP) and false negative (FN) were present leading to an accuracy of 69.71% (CI 95%: 62.54–76.04), specificity of 59.30% (CI 95%: 48.74–69.07) and sensitivity of 79.78% (CI 95%: 70.28–86.81) (Table 2). These results highlight the need to develop more complex approaches using AI models to distinguish short-term from long-term survivors.

**Table 2:** Patient-level performance of non-AI and AI models for the prediction of survival outcome.

Model	Area under the curve (AUC)	Specificity (%)	Sensitivity (%)	Accuracy (%)	Precision (%)	F1 (%)
Surgery type only (non-AI)	/	59.30 (48.74–69.07)	79.78 (70.28–86.81)	69.71 (62.54–76.04)	66.98 (57.57–75.20)	72.82 (65.22–79.38)
Two-class model: clinical data only	0.826 (0.714–0.915)	82.86 (69.70–94.12)	65.71 (50.00–81.40)	74.29 (62.86–84.29)	79.31 (62.50–92.86)	71.88 (57.63–83.58)
Two-class model: MRI only	0.895 (0.775–0.9848)	94.44 (81.82–1.00)	68.42 (47.06–88.89)	81.08 (67.57–91.89)	92.86 (76.92–1.00)	78.42 (60.61–92.31)
Two-class model: MRI only and clinical data	0.864 (0.731–0.971)	83.33 (64.71–100.00)	85.00 (66.67–100.00)	84.21 (71.05–94.74)	85.00 (68.74–100.00)	85.00 (70.97–95.24)
Three-class model: MRI only	0.948 (0.882–0.992)	93.59 (88.03–98.41)	87.18 (76.68–96.97)	87.18 (76.92–97.44)	87.74 (77.09–96.50)	97.12 (75.09–96.08)
Three-class model: MRI only and clinical data	0.925 (0.849–0.988)	92.31 (86.07–97.53)	84.62 (72.54–95.24)	84.62 (71.79–94.87)	82.05 (69.23–92.31)	84.60 (71.79–94.84)
Four-class model: MRI only	0.907 (0.816–0.971)	90.46 (85.75–94.93)	71.67 (55.83–85.28)	71.05 (55.26–84.21)	75.39 (62.03–87.22)	71.33 (54.17–83.96)
Four-class model: MRI only and clinical data	0.908 (0.835–0.975)	92.06 (87.51–96.23)	76.11 (61.63–88.75)	76.32 (63.16–89.47)	80.63 (68.06–91.18)	76.82 (61.33–88.30)

Values are point estimates with 95% confidence intervals. For the surgery-type-only (non-AI) approach, CIs were obtained using the Wilson score method; for AI models, CIs were obtained by patient-level bootstrapping (2,000 resamples). For multiclass tasks, metrics were computed per class (one-vs-rest) and macro-averaged across classes.

#### Deep learning model based only on pre-chemoradiotherapy MRI

##### Patient-level results

As part of the clinical workflow for radiotherapy process, pre-chemoradiotherapy T1-Gd MRI is always performed and accessible in clinical routine (25). For this reason, as presented in Figure 3a, we firstly used this unique input to develop deep learning model able to distinguish, before the initiation of radiotherapy and chemotherapy treatment, short (OS < 332 days) to long-survivor (OS ≥ 332 days) patients. Binary deep learning model presented good performance with AUC = 0.89 (CI 95%: 0.775–0.985) (Figure 3b) and, as shown by the confusion matrix in Figure 3c, relatively low numbers of FP and FN were obtained resulting in an accuracy, specificity and sensitivity of 81.08% (CI 95%: 71.05–94.74), 94.44% (CI 95%: 81.82–1.00) and 68.42% (CI 95%: 47.06–88.89) respectively (Table 2).

**Figure 3:**
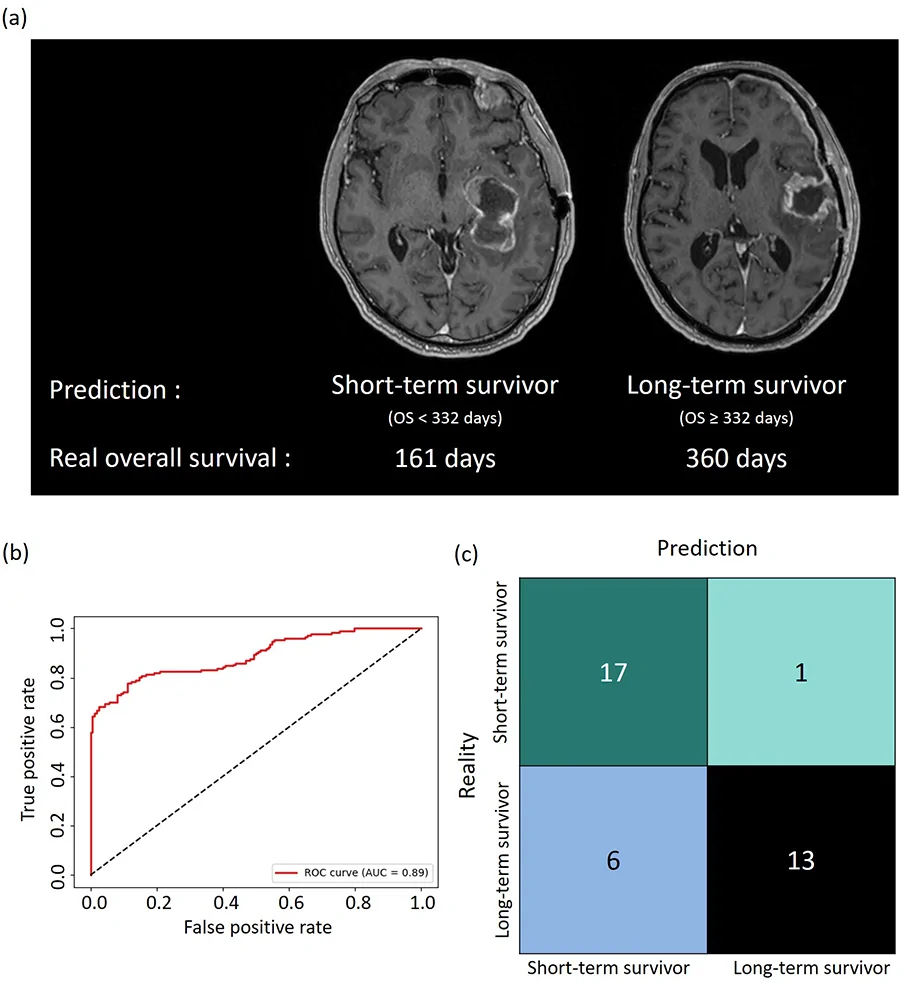
Patient-level performance of the MRI-only two-class deep learning model. **(a)** Representative MRI of short (OS < 332 days) and long-term survivors (OS ≥ 332 days) correctly predicted by the deep learning model. **(b)** ROC curve and **(c)** confusion matrix of the deep learning model. OS, overall survival; ROC, receiver operating characteristic; AUC, area under the ROC curve.

##### Slice-level results

For slice-level results, we evaluated the standalone deep learning model on individual MRI slices as a secondary analysis. The performance metrics demonstrated consistent predictive trends compared to the primary patient-level aggregation and are comprehensively detailed in Supplementary Table S3.

### Prediction of three classes overall survival outcome

#### Deep learning model based only on pre-chemoradiotherapy MRI

##### Patient-level results

To challenge the AI model and to assess whether the model could discriminate more granular survival categories, we evaluated the ability of another model to predict more classes of survival outcome. Three classes were defined as follow the terciles method: class 1 (OS ≤ 202 days), class 2 (202 days < OS ≤ 420 days) and class 3 (OS > 420 days). Performance evaluation of the three classes model of slice-level achieved an accuracy of 87.18% (CI 95%: 76.92–97.44), specificity of 93.59% (CI 95%: 88.03–98.41), and sensitivity of 87.18% (CI 95%: 76.70–96.30) (Table 2).

##### Slice-level results

Performance evaluation of the three classes model of slice-level achieved an accuracy of 81.77% (CI 95%: 70.66–91.45), specificity of 90.88% (CI 95%: 85.25–95.74), and sensitivity of 81.77% (CI 95%: 71.07–91.33) (Supplementary Table S3).

#### Deep hybrid learning based on pre-chemoradiotherapy MRI and clinical data

Integrating clinical variables such as WHO performance status, age, sex, and surgery type was hypothesized to enhance the predictive performance of the AI models. For this reason, we developed a DHL model combining the pre-chemoradiotherapy MRI deep learning model prediction and classification machine learning model including clinical data (Figure 1). After performance evaluation and hyperparameters optimization, *RandomForestClassifier* has shown to be the more suitable model.

Three representative patients from each class are shown in Supplementary Figure S3a. As shown in Supplementary Figure S3b, the three classes model using pre-chemoradiotherapy MRI and clinical data presented AUC with 0.96 for class 1, 0.93 for class 2, and 0.92 for class 3. Performance evaluation of the three classes model reached an accuracy of 84.62% (−2.56% in comparison to the pre-chemoradiotherapy MRI only deep learning model at patient-level) (CI 95%: 71.79–94.87), specificity of 92.31% (−1.28%) (CI 95%: 86.07–97.53), and sensitivity of 84.62% (−2.56%) (CI 95%: 72.54–95.24) (Supplementary Figure S3c and Table 2).

At patient-level, the MRI-only DL model correctly classified 34 of 39 patients (87.18%), whereas the multimodal DHL model correctly classify 33 patients of 39 patients (84.62%). The two models agreed on 38 of 39 patients (33 concordant correct, 5 concordant incorrect, 1 discordant pairs). The exact McNemar test was therefore non-significant (*p* = 1.00), indicating no difference in patient-level accuracy between the two models. Paired patient-level bootstrap comparisons (2,000 resamples) further confirmed that adding clinical data provided no statistically significant improvement across continuous or discrete metrics: macro-averaged AUC was 0.920 for DHL versus 0.948 for DL alone (Δ= −0.028, 95% CI: [−0.0734; +0.0123], *p* = 0.166), sensitivity was 84.62% versus 87.18% (Δ= −0.026, 95% CI: [−0.0833; +0.0000], *p* = 0.724), and specificity was 92.31% versus 93.59% (Δ= −0.013, 95% CI: [−0.0417; +0.0000], *p* = 0.724).

To further understand the importance of each variable in the DHL model prediction, feature importance of the model was evaluated and is shown in Supplementary Figure S4a. Pre-chemoradiotherapy MRI feature importance was far higher (65%) than other clinical data (age, surgery type, WHO performance status and sex 16%, 12%, 6%, and 1% respectively).

### Prediction of four classes overall survival outcome

#### Deep hybrid learning based on pre-chemoradiotherapy MRI and clinical data

Finally, due to satisfying performance in the three classes AI model, we used a similar DHL architecture to develop a four classes model. As shown earlier, Figure 4a showed four representative brain MRI patients, which were hardly distinguishable in terms of survival outcomes by humans. Following a performance evaluation and hyperparameter optimization, the *RandomForestClassifier* model was identified as the most appropriate model.

**Figure 4:**
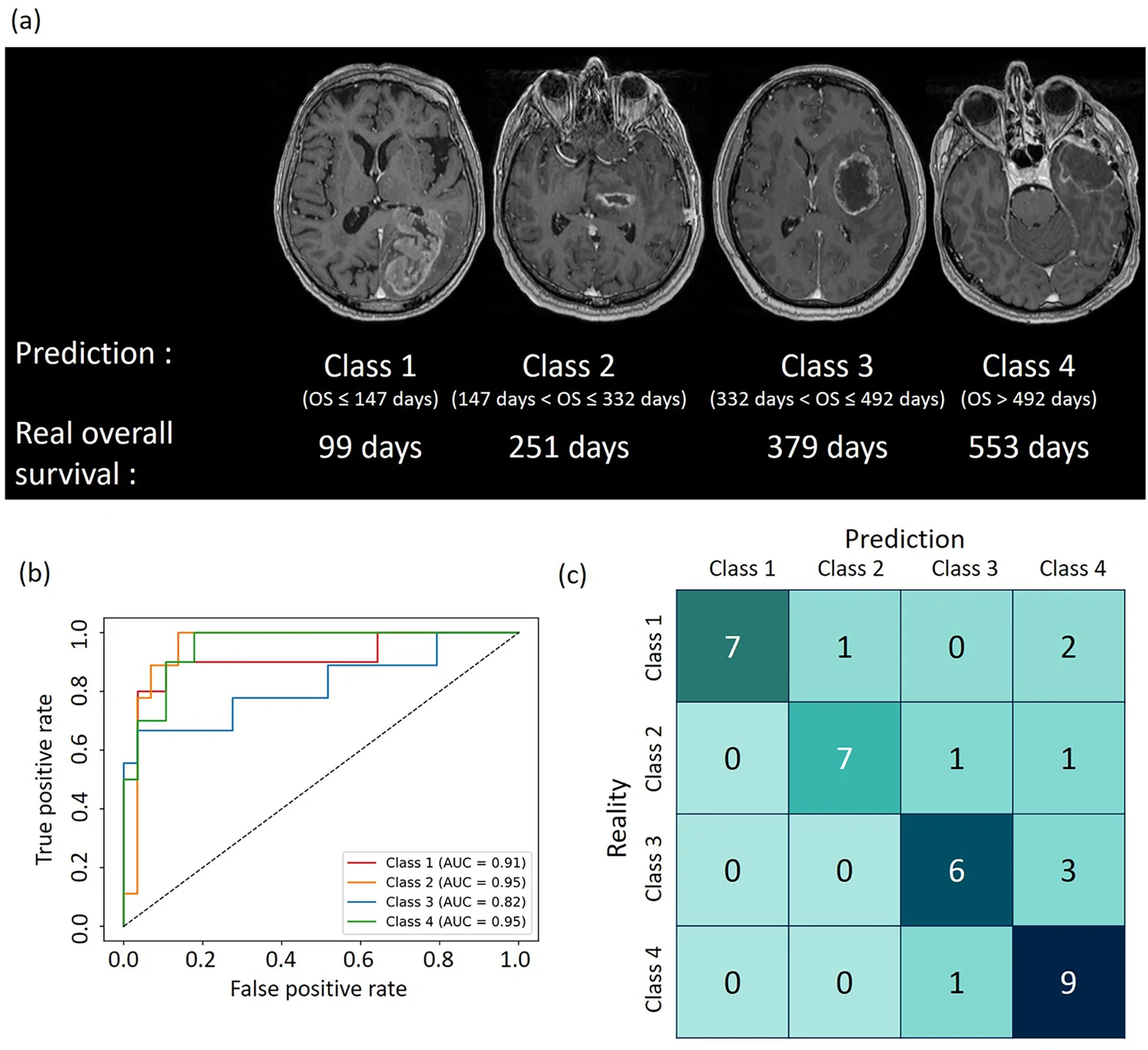
Performance of the four-class deep hybrid learning model using MRI and clinical data. **(a)** Representative MRI of class 1 (OS ≤ 147 days), class 2 (147 days < OS ≤ 332 days), class 3 (332 < OS ≤ 492 days) and class 4 (OS > 492 days) correctly predicted by the deep learning model. **(b)** One-vs-rest ROC curve for each class and **(c)** confusion matrix of the deep learning model. OS, overall survival; ROC, receiver operating characteristic; AUC, area under the ROC curve.

However, as shown by Figures 4b, c and Table 2, the four classes DHL model presented high AUC of 0.91, 0.96, 0.91, and 0.91 for class 1–4 respectively. Accuracy, specificity and sensitivity were 76.32% (CI 95%: 63.16–89.47), 92.06% (CI 95%: 87.51–96.23), and 76.11% (CI 95%: 61.63–88.75) respectively. Supplementary Figure S4b illustrates the feature importance for this model. The analysis revealed that pre-chemoradiotherapy MRI was the most critical predictor (67.3%), followed by age (13.5%), surgery type (13%), WHO performance status (5.2%), and sex (1%).

## Discussion

In clinical practice, the response to treatment for glioblastomas is typically assessed two months after the completion of radiotherapy and chemotherapy on anatomical MRI. However, this delay is a significant constraint, particularly due to the rapid progression of the disease and the short median survival rate of patients. Therefore, the implementation of a tool that could predict survival outcome earlier could have a substantial clinical impact (15). In this study, we developed AI models able to predict two, three and four classes of survival outcome using firstly only pre-chemoradiotherapy MRI and then combining with pre-chemoradiotherapy clinical data (WHO performance status, age, sex, and surgery type). The data analyzed in this study were exclusively obtained from routinely collected clinical records, with no molecular data incorporated. This choice was motivated by our aim to develop a model relying solely on information available in clinical routine before radiotherapy. However, their absence may affect both model performance and interpretation, as these factors are known to influence survival in glioblastoma (MGMT methylation status, in particular) and carry prognostic information not captured by our clinical and imaging data. Therefore, their integration represents a valuable direction for future work.

Literature indicates that the type of surgery is a variable strongly associated with therapeutic response (24), as we shown in this article and in Supplementary Figure S5 with five representative patients with different overall survival, non-AI surgery type-based model is not efficient enough to predict overall survival classification. Indeed, glioblastoma is a tumor characterized by significant spatial and intra tumoral heterogeneity (26–28). This heterogeneity is primarily defined by vascularization, edema, necrosis and tumor infiltration in different regions of the tumor (29). MRI can be used to characterize the entire tumor and integrate spatial information. Clinical data and MRI are complementary because they offer different perspectives on the therapeutic response. In this article, we evaluated the performance of overall survival prediction across three classification tasks (binary, three, and four classes). While the integration of clinical data into MRI did not uniformly improve the AUC across all tasks compared to MRI alone (Table 2), it fundamentally enhanced the model's predictive certainty. Notably, for the three-class task, the multimodal approach significantly increased the model's confidence in identifying the correct survival class (*p* < 0.001).

Surgery type and resection volume could have been expected to be an important factor in predicting overall survival. One possible explanation is that surgery type is partly redundant with imaging, since post-surgical changes (the resection cavity, for example) are visible on MRI and may already be captured by the deep learning model. To confirm this hypothesis, future work would require explainability analyses sur as saliency maps.

We acknowledge that while 1,575 MRI slices were analyzed, our effective independent sample size is limited to 175 patients. Consequently, expanding the dataset with additional patients is necessary to robustly validate our findings, particularly for the three- and four-class models where the test cohort is relatively small.

In the literature, several studies have focused on predicting the survival of glioblastoma patients based on pre-chemoradiotherapy data. However, most of them used publicly available datasets (9, 30–32). Although the use of these data allows good reproducibility and easy comparison of models between studies, they do not reflect standard clinical data. Publicly available datasets are generally well structured and organized, which may differ from the discordance often seen in routine clinical data and the variability of clinical practices (33). Yang et al. (30) used the Medical Image Computing and Computer Assisted Intervention (MICCAI) Brain Tumor Segmentation (BraTS) challenge 2020 (34–36) which includes four imaging sequences (T1, T1ce, T2 and T2-FLAIR) and performed a two-class survival classification with an accuracy and AUC of 78.3% and 0.86 and 65.2% and 0.81 for the three-class model. One study (37) used four imaging sequences (T1c, T1, T2, and T2-FLAIR) and clinical data from 125 patients to predict survival of glioblastoma in two classes (< 12 months, and > 12 months). The authors developed three machine learning algorithms based on radiomic features. The best results among these algorithms were obtained by the support vector machine (SVM) model, with an AUC of 0.75 in the validation cohort. These features were then combined into a radiomics nomogram that reached an AUC of 0.919 in the validation cohort. While these radiomics-based approaches show promising performance, our study explores a different strategy based on newly developed deep learning methodologies. However, direct comparison is limited, as performance metrics were obtained on different cohorts and with different survival endpoints. In another study (38), a deep learning model was used to classify the survival of 133 patients into three classes (<1 year, 1–2 years, >2 years) with structural images (T1w, T2w, T2-FLAIR), resting state functional MRI and clinical features. The model performed well, classifying survival with an accuracy of 90.6% with MRI and clinical data. The performance of our model is inferior to the results reported in the article by Luckett et al*.,* with an accuracy of 87%. However, a direct comparison is limited, as their model relied on multimodal pre-operative data, including resting-state fMRI, cortical structural features, and demographics, whereas our approach used anatomical MRI. More broadly, other studies have addressed survival prediction using different types of data: some have explored non-anatomical imaging modalities, such as amino-acid (18F-FET) PET radiomics (39), while others have relied on clinical and molecular variables alone, without imaging (40). However, in our study we have chosen to use clinical and imaging data that are regularly collected as part of standard clinical practice to enable potential rapid implementation in the clinical workflow. Ellingson et al*.* (39) conducted highly valuable work by demonstrating the prognostic significance of postoperative residual contrast-enhancing tumor volume. While their findings establish a crucial clinical foundation, developing a deep learning model to predict patient overall survival builds upon this interest by addressing several methodological and practical limitations. Indeed, whereas conventional statistical models rely on macroscopic metrics (cm^3^) to stratify patients, AI models are capable of extracting underlying features invisible to the human eye, such as spatial heterogeneity and subtle tissue textures. Furthermore, manual volumetric assessment in standard practice remains time-consuming and subject to substantial inter-observer variability. As a fully automated method, our approach could offer more reproducible, less operator-dependant measurements. Moreover, integrating imaging with clinical data represents a step toward turning this biomarker into a clinically usable prognostic tool, though further validation is required.

Additionally, other studies (13, 40) have also been interested in predicting patient survival with glioblastomas, but using data obtained during and after treatment. However, the data used for prediction were collected after the start of treatment, meaning that patients may therefore be subject to potential side effects related to their therapy.

Although our study has demonstrated satisfactory results, it is important to emphasize that the developed tool cannot replace the professional judgement of a radiation oncologist in the therapeutic management of a patient. This tool could provide the radiation oncologist with additional prognostic information at the pre-treatment stage, complementing the overall clinical assessment (41).

In our study, we did not perform external validation of the model. Indeed, publicly available glioblastoma datasets (42, 43) with overall survival data provide only diagnostic (preoperative) imaging. Although LUMIERE (44) annotates surgical time points (pre- and post-operative), it does not indicate the start of radiotherapy. While the post-operative MRI could serve as an approximation of the pre-chemoradiotherapy time point, the interval between surgery and the initiation of chemoradiotherapy varies across patients and is typically not documented. However, the objective of our study is to predict survival classification in order to optimize radiotherapy treatment. Therefore, our model must be used with MRI obtained approximately 13.50 days before radiotherapy. Glioblastoma is a highly aggressive tumor, consequently, pre-chemoradiotherapy MRI characterizes the lesion at the time of treatment planning, whereas MRI diagnostic is performed several weeks earlier (40 days). Furthermore, the extent of resection is shown in the literature and in our study to be a prognostic factor for survival. Pre-chemoradiotherapy MRI contains information on the extent of resection. Consequently, using a published dataset would therefore have introduced significant temporal bias due to the rapid tumor progression.

External validation of our fixed model on independent multi-center cohorts is a priority for future work. This would simply require deploying the trained algorithm locally at partner centers with harmonized acquisition protocols and documented treatment timing, ensuring a standardized pre-chemoradiotherapy time point across sites. Furthermore, given the privacy and data-sharing constraints that currently limit the pooling of medical imaging across institutions, federated learning represents a promising strategy for future multicenter model development. This approach would allow for the collaborative training of more robust and generalizable models without ever transferring sensitive patient data outside individual institutions.

One of the main limitations of our study is the use of 2D slices instead of a 2.5D or a 3D volume. This decision was adopted to reduce the computational time required by the model. However, the use of a 2.5D or a 3D model could potentially improve the performance of the model (45, 46). These methods can capture intra tumoral heterogeneity. Additionally, we did not evaluate models with more than four classes because increasing the number of classes further reduces the already limited number of patients per class. The focus on deceased patients represents a methodological limitation that substantially reduces the data. This restriction stems from our multiclass classification framework, which requires deterministic survival labels. While some right-censored patients could theoretically be assigned an unambiguous label (e.g., a patient censored beyond a specific time threshold definitively belongs to the upper survival class), we chose to exclude all censored patients to maintain a strictly identical patient cohort across our two-, three- and four-class models. Retaining unambiguous censored patients would have resulted in varying cohort sizes and compositions depending on the specific time thresholds of each classification task, complicating comparisons between models. Beyond reducing the sample size, this exclusion may introduce a selection bias, as excluded patients may differ from the analyzed cohort, limiting generalizability. Future work could adopt survival-analysis approaches that account for censoring (Cox models or deep survival models for example), enabling the full cohort to be analyzed without exclusion. It is worth noting that under the updated WHO 2021 classification, one tumor in our cohort originally classified as glioblastoma (WHO 2016) corresponds to an Astrocytoma, IDH-mutant, CNS WHO grade 4. To assess whether this diagnostic update affect our results, we performed a dedicated sensitivity analysis. Excluding this single case at the meta-classifier level (without retraining the deep learning backbone) produced only a small change in performance (accuracy of 85%), indicating that its inclusion did not materially affect performance. Therefore, the patient was retained to preserve the integrity of the original study design (*n* = 175). Another limitation of our study is the absence of digital subtraction between pre and post-contrast T1-weighted images. While we acknowledge that this technique is essential to accurately isolate the true enhancing signal from surgical alterations and is strongly correlated with therapeutic response (47, 48), it is not performed in standard clinical routine at our center. Future prospective studies should consider integrating these subtraction maps. A further limitation lies in the methodology used to integrate imaging and clinical data. More recent methods with deep multimodal fusion could improve the model performance (49, 50). Furthermore, a methodological limitation of our deep hybrid learning framework lies in the training of the meta-classifier. The deep learning probabilities provided as inputs to the second-step meta-classifier were generated in-sample (from the same patients used to train the first-step deep learning model) rather than through a rigorous out-of-fold cross-validation scheme. This in-sample generation can introduce an optimistic bias, as the meta-classifier is trained on highly confident deep learning predictions. While our strict evaluation on a completely independent test set demonstrated that the models successfully generalized, this two-stage training design remains theoretically sub-optimal. Future prospective studies deploying similar stacked architectures should generate out-of-fold deep learning predictions to optimally calibrate the meta-classifier. Additionally, this study focused on assessing survival outcomes at the pre-chemoradiotherapy stage. A logical and valuable future perspective would be the integration of spatial radiotherapy dose distributions (dose map or dosiomics features) to capture the impact of the actual delivered treatment on patient survival (51, 52).

## Conclusion

In this study, we introduce a deep learning model for predicting overall survival in glioblastoma patients, leveraging pre-chemoradiotherapy MRI alongside clinical data. The proposed workflow was developed using only clinical routine data, yielding promising results. The proposed model showed the potential to provide early prognostic stratification in glioblastoma, which could complement clinical assessment. However, these preliminary results require external validation in independent cohorts before any clinical application.

## Data Availability

The original contributions presented in the study are included in the article/Supplementary Material, further inquiries can be directed to the corresponding author.
