# Supplementary material for: Deep learning prediction of overall survival in glioblastoma patients using pre-chemoradiotherapy MRI and clinical data
Source: Front Radiol. 2026 Oct 13;6:1963539. doi: 10.3389/fradi.2026.1963539 (PMC13710324; doi:10.3389/fradi.2026.1963539)
Supplement: Supplementary file 1 [file Datasheet1.pdf]

Supplementary materials and methods 1 : ResNet-51Q architecture

In our study, we chose to use an AI method based on a network architecture derived from ResNet-50 [1]. In order to increase efficiency and learning capacity, the ResNet-51Q [2] network made several significant changes to the ResNet-50 architecture. It has a bottleneck-like structure similar to ResNet-50, but its convolutional operations were improved. Instead of the conventional max pooling layer present in ResNet-50, it starts with a quadruple convolutional stem with two activations. In contrast to ResNet-50, which has four main blocks with depths of 3, 4, 6, and 3, the architecture has four main blocks with depths of 2, 4, 6, and 4. One significant distinction is the convolutional approach: ResNet-51Q uses grouped convolutions with a group size of 32 and a bottleneck ratio of 0.25 in the first three blocks, whereas ResNet-50 mainly uses standard convolutions. Depth wise convolutions with a ratio of 1.0 are incorporated in the final block to further improve efficiency. In contrast to ResNet-50, which reaches 2048 channels in its final stage, the number of channels rises from 256 to 512, then to 1536, which stays constant in the final block. Furthermore, ResNet-51Q enhances gradient propagation and convergence by substituting the SiLU (Sigmoid Linear Unit) for the conventional ReLU activation of ResNet-50. The network ends with a 1536 feature layer that is already fully connected. ResNet-51Q is better suited for contemporary deep learning applications that demand both high performance and resource efficiency thanks to these architectural improvements, which optimize the trade-off between depth, computational efficiency, and representational power.

[1] K. He, X. Zhang, S. Ren, et J. Sun, « Deep Residual Learning for Image Recognition », in *2016 IEEE Conference on Computer Vision and Pattern Recognition (CVPR)*, Las Vegas, NV, USA: IEEE, juin 2016, p. 770-778. doi: 10.1109/CVPR.2016.90.

[2] R. Wightman *et al.*, *rwightman/pytorch-image-models: v0.8.10dev0 Release*. (7 février 2023). Zenodo. doi: 10.5281/ZENODO.4414861.

Supplementary Figure 1

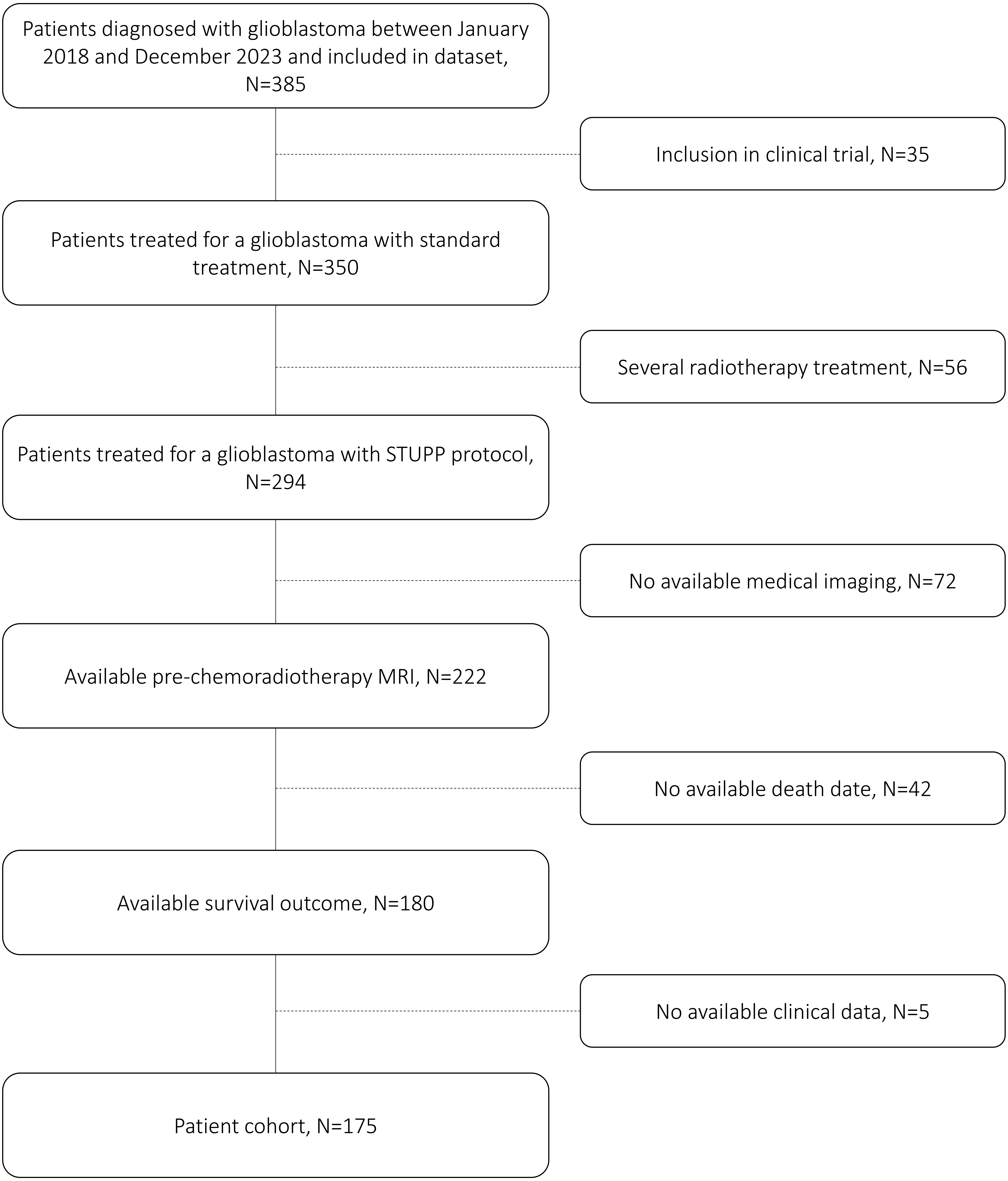

Supplementary figure 1: Patient cohort

Supplementary Figure 2

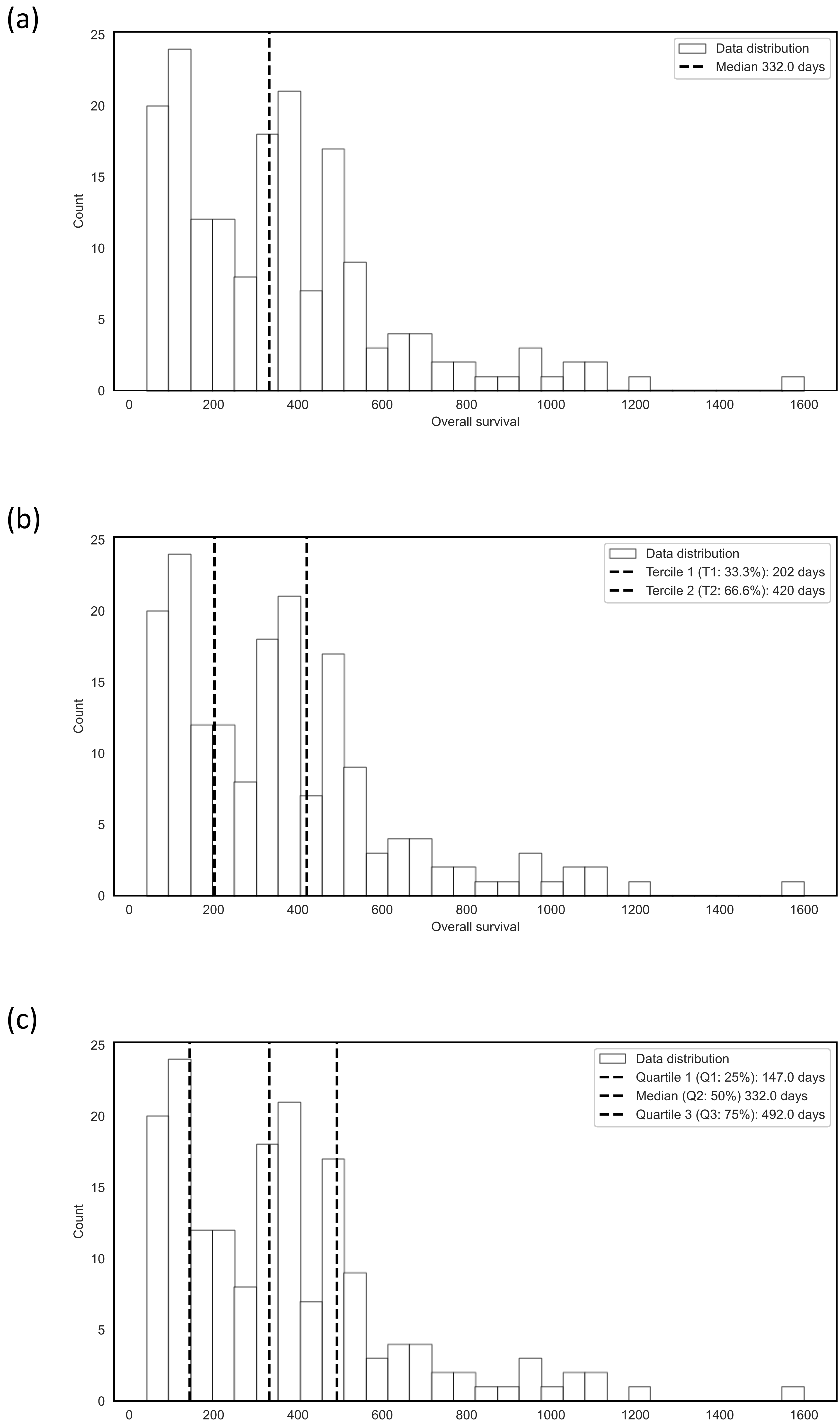

Supplementary figure 2: Overall survival histograms including: (a) median; (b) terciles and (c) quartiles.

Supplementary Figure 3

(a)

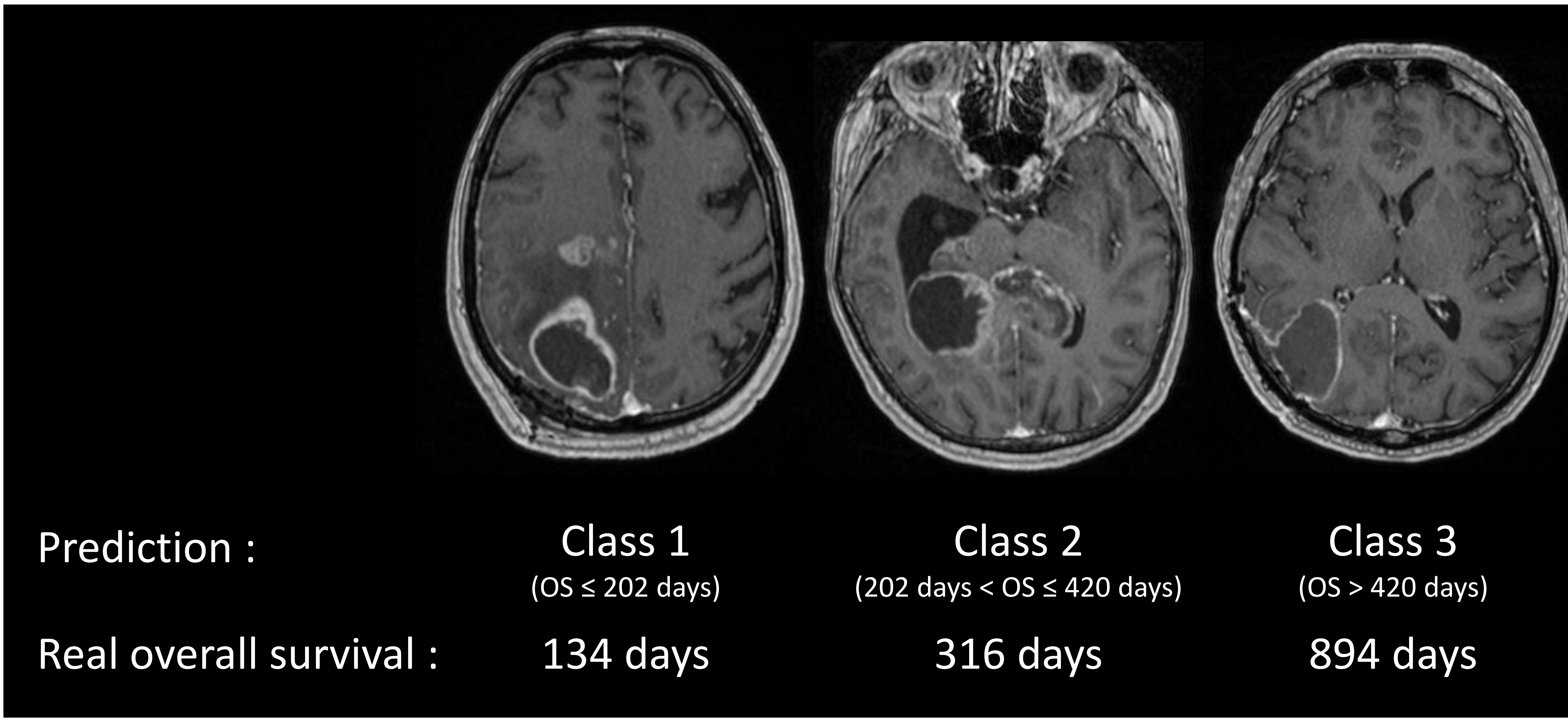

(b)

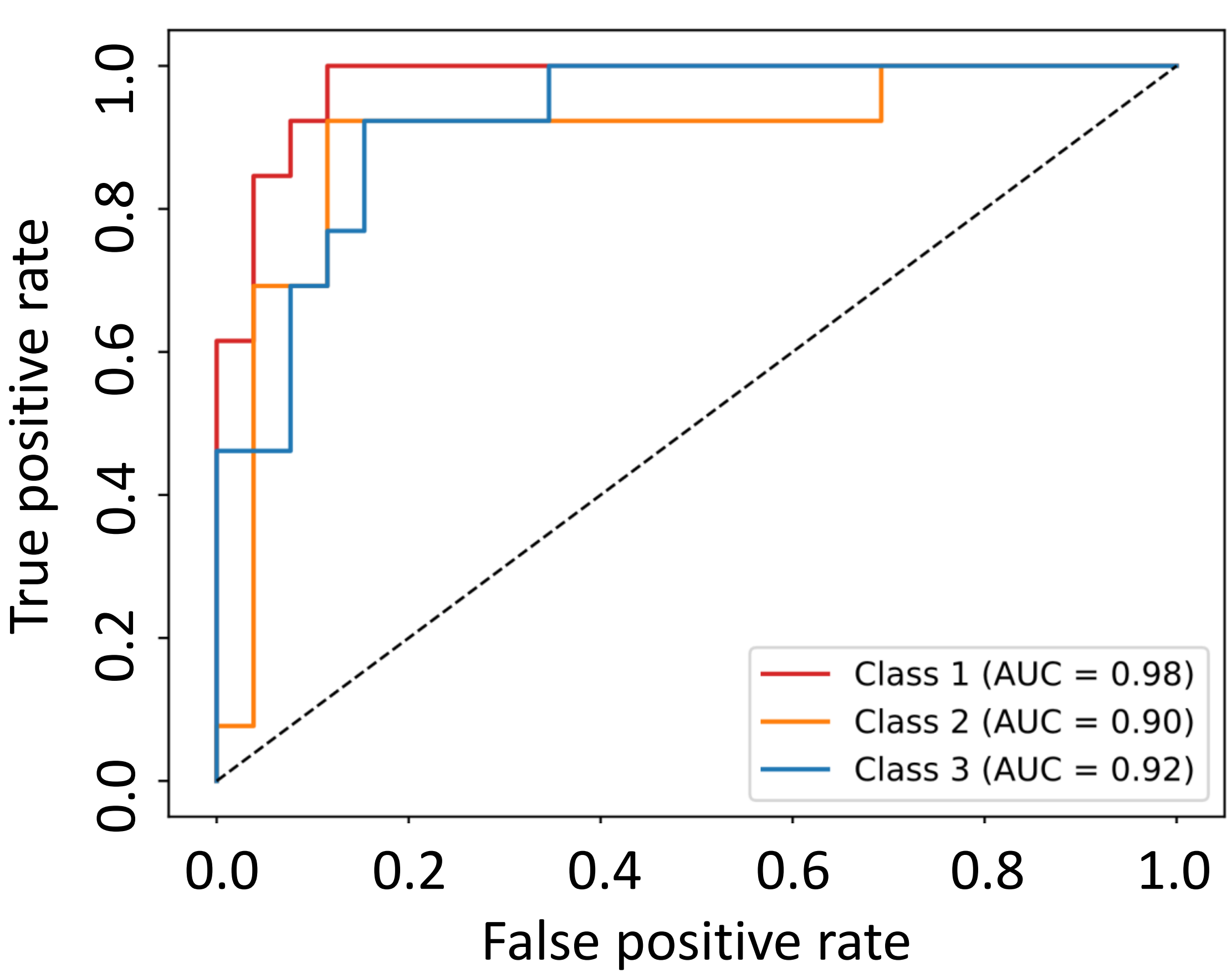

(c)

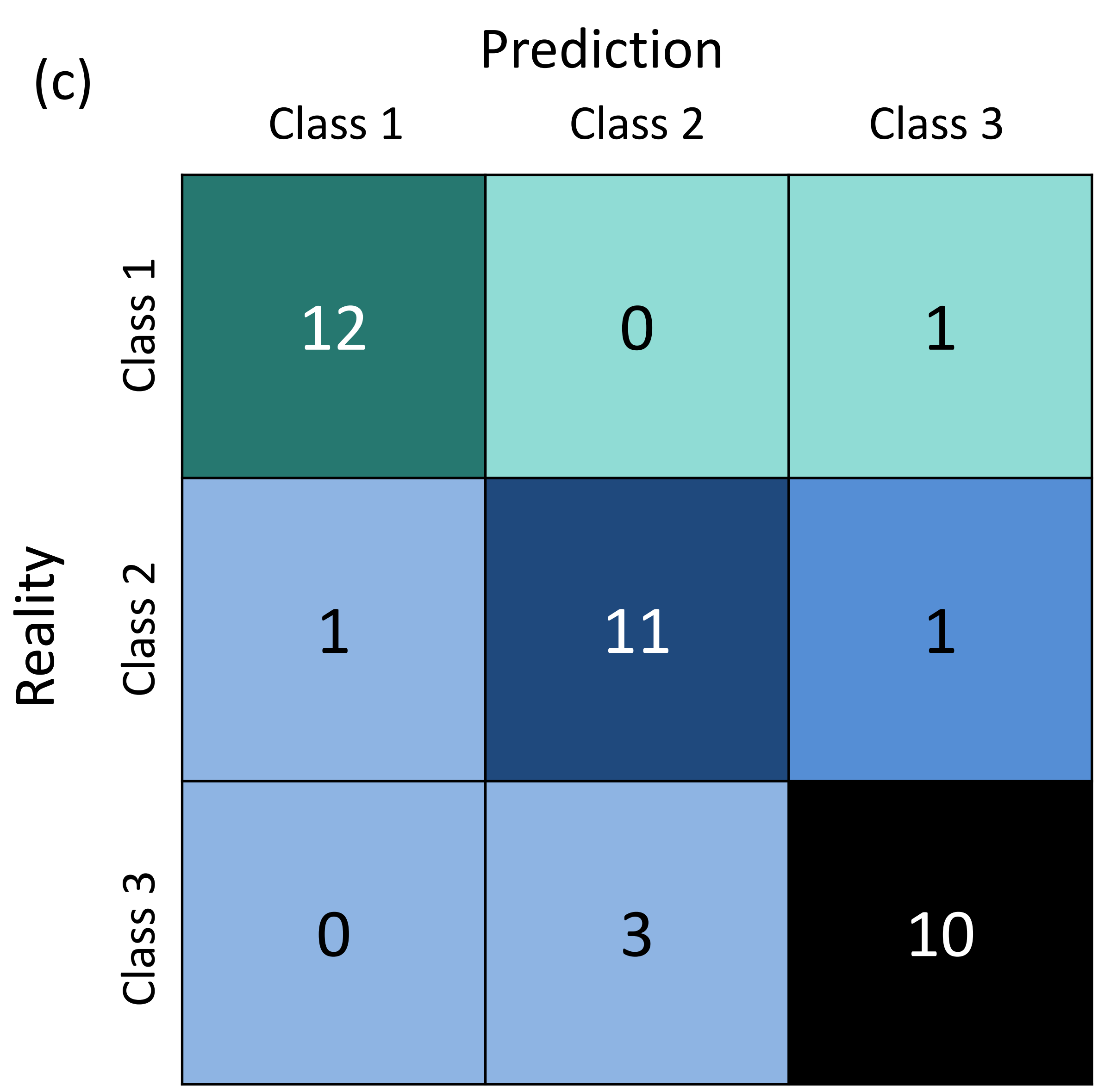

Supplementary Figure 3: Performance of the three-class deep hybrid learning model using MRI and clinical data. (a) Representative MRI of class 1 (OS ≤ 202 days), class 2 (202 days < OS ≤ 420 days) and class 3 (OS > 420 days) correctly predicted by the deep hybrid learning model. (b) One-vs-rest ROC curve for each class and (c) confusion matrix of the deep hybrid learning model.

OS: Overall Survival; ROC: Receiver Operating Characteristic; AUC: Area Under the ROC Curve

Supplementary Figure 4

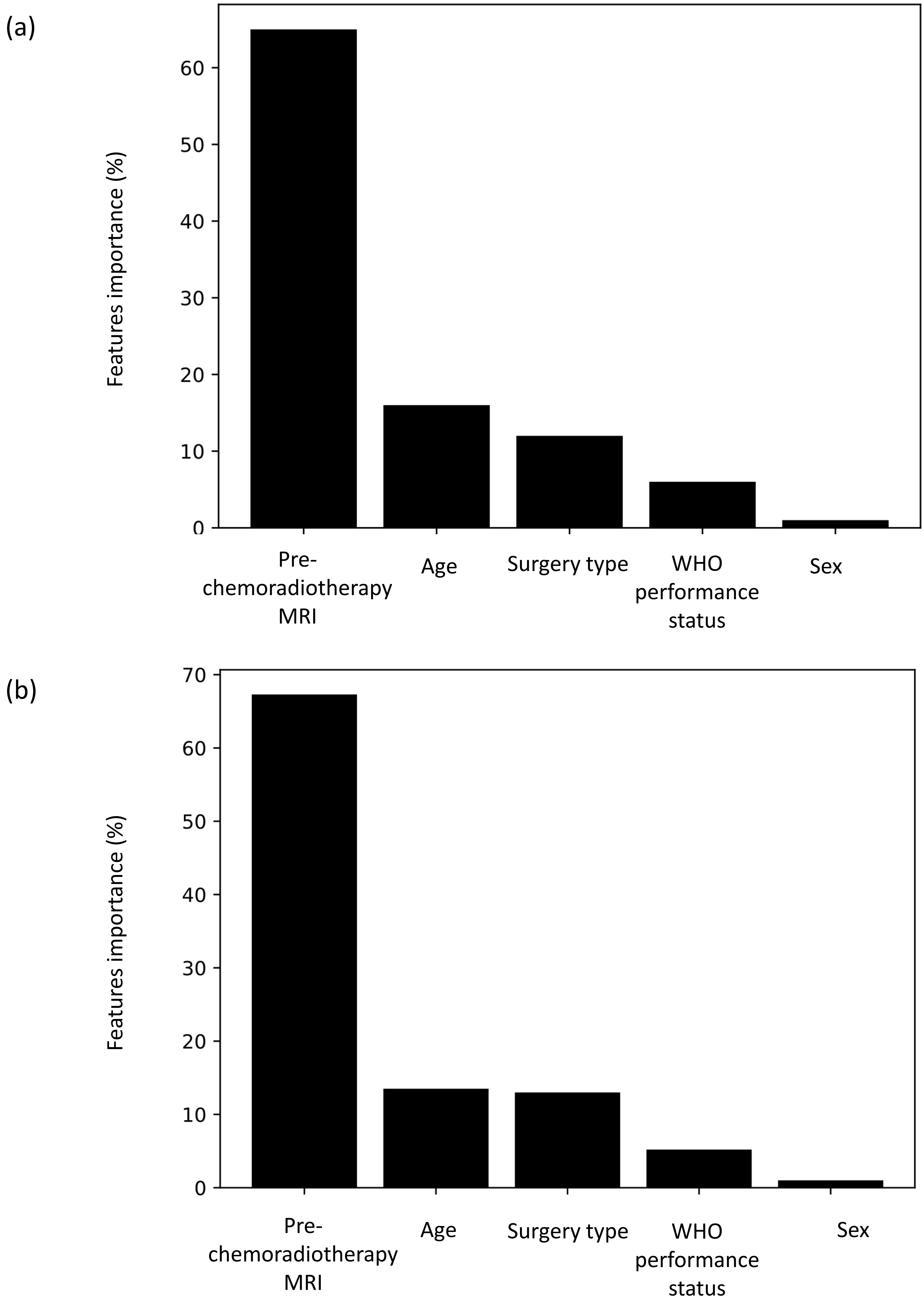

Supplementary figure 4: Importance of the features for survival outcome prediction in the (a) three and (b) four classes models.

Supplementary Figure 5

	Patient 1	Patient 2	Patient 3	Patient 4	Patient 5
**Pre-treatment MRI**	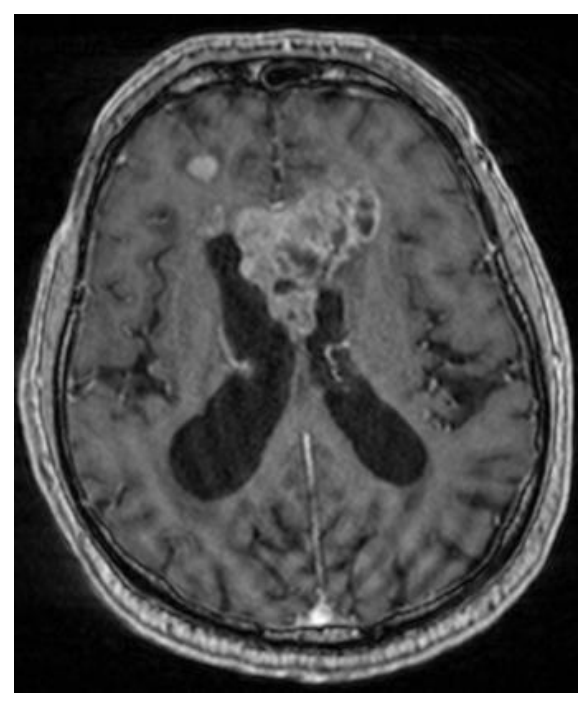	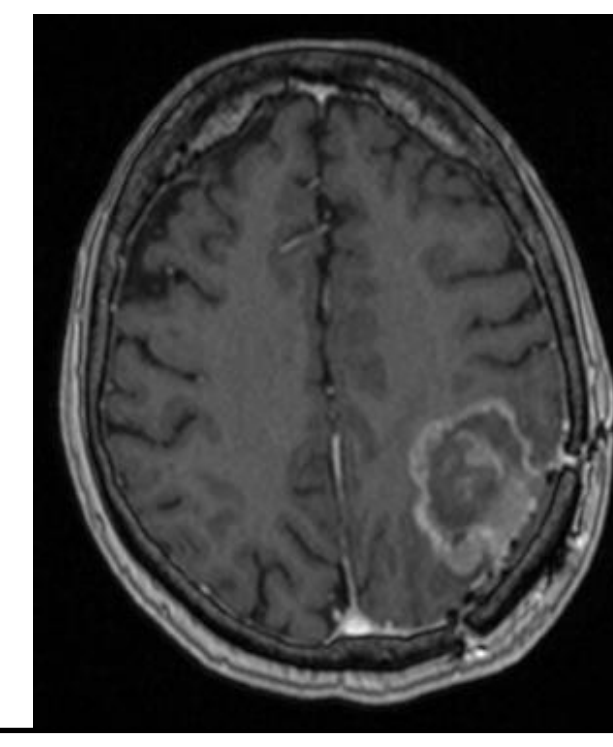	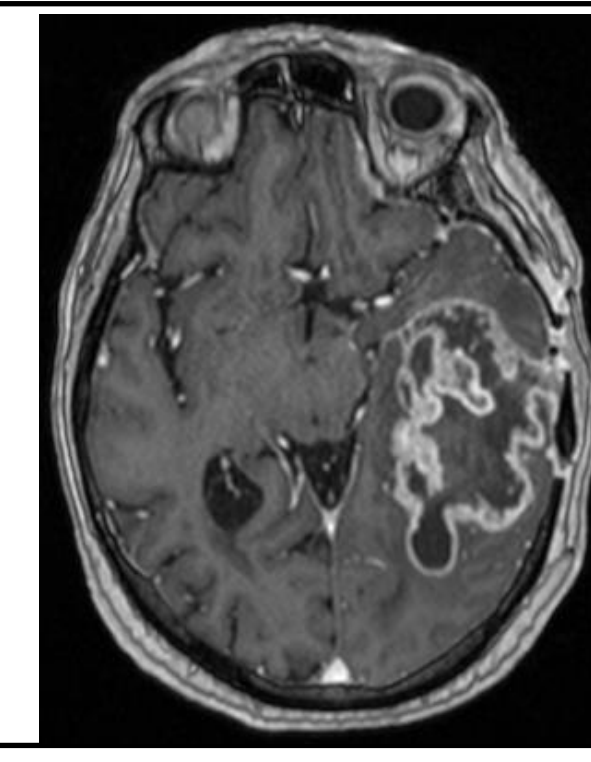	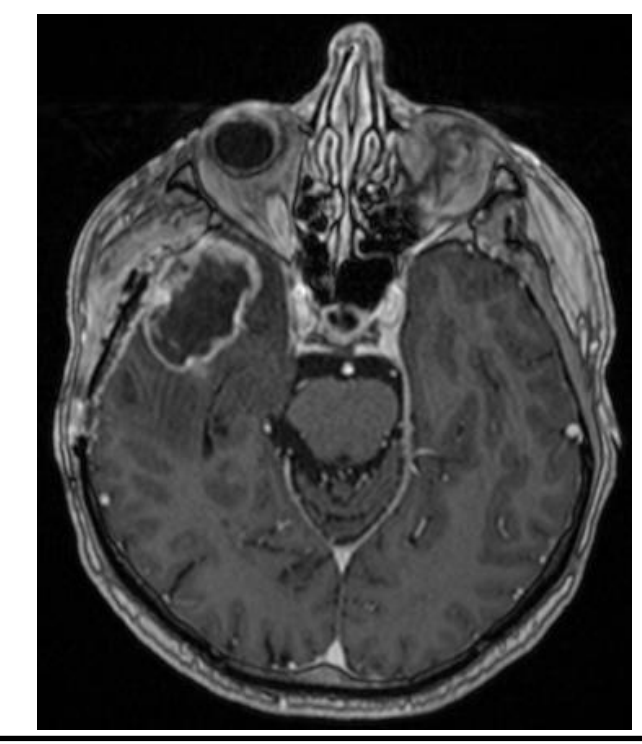	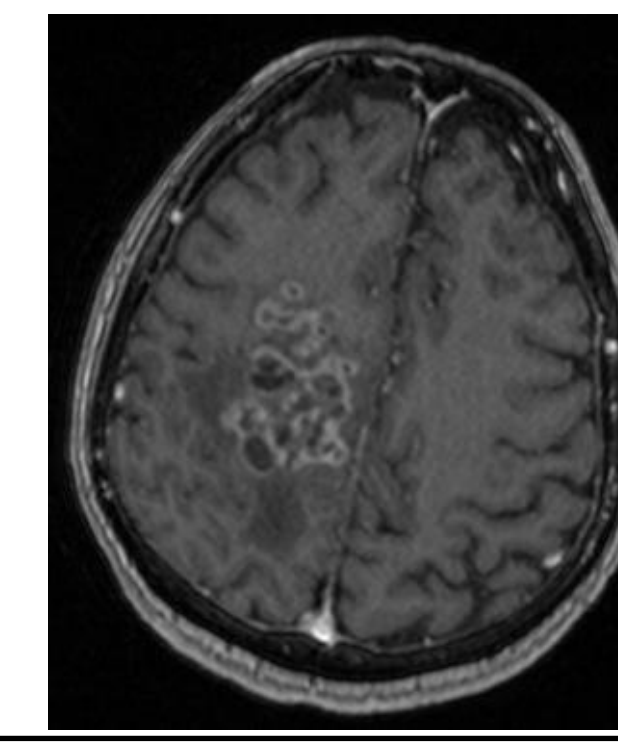
**Clinical data**	Age: 86.92 years<br>Surgery type: Biopsy<br>WHO status performance: 1<br>Sex: Male	Age: 74.80 years<br>Surgery type: Incomplete<br>WHO status performance: 0<br>Sex: Female	Age: 83.72 years<br>Surgery type: Biopsy<br>WHO status performance: 1<br>Sex: Female	Age: 65.26 years<br>Surgery type: Complete<br>WHO status performance: 1<br>Sex: Male	Age: 66.15 years<br>Surgery type: Biopsy<br>WHO status performance: 2<br>Sex: Female
**Surgery type only (non-AI)**<br>(overall survival prediction)	OS < 332 days	OS ≥ 332 days	OS < 332 days	OS ≥ 332 days	OS < 332 days
**Two-class model: MRI only**<br>(overall survival prediction)	OS < 332 days	OS ≥ 332 days	OS < 332 days	OS ≥ 332 days	OS ≥ 332 days
**Three-class model: MRI only**<br>(overall survival prediction)	OS ≤ 202 days	202 days < OS ≤ 420 days	OS ≤ 202 days	OS > 420 days	OS > 420 days
**Three-class model: MRI only and clinical data**<br>(overall survival prediction)	OS ≤ 202 days	202 days < OS ≤ 420 days	OS > 420 days	OS > 420 days	OS > 420 days
**Four-class model: MRI only and clinical data**<br>(overall survival prediction)	OS ≤ 147 days	147 days < OS ≤ 332 days	332 days < OS ≤ 492 days	OS > 492 days	OS > 492 days
**Real overall survival**	106 days	213 days	490 days	545 days	800 days

Supplementary Figure 5: Examples of pre-chemoradiotherapy MRI and clinical data and corresponding prediction of survival outcome for each models. Underline cases correspond to non accurate prediction.

Supplementary table 1: : Description of the patient cohort for three-class survival outcome prediction.

Category	All Patients (n=175)	Class 1 (n=59)<br>OS ≤ 202 days	Class 2 (n=58)<br>202 days < OS ≤ 420 days	Class 3 (n=58)<br>OS > 420 days
**Survival (days)**	mean: 363.4<br>median: 332.0 (IQR: 349.0)<br>min-max: [42.0-1600.0]	mean: 113.9<br>median: 116.0 (IQR: 76.0)<br>min-max: [42.0-202.0]	mean: 321.1<br>median: 332.0 (IQR: 80.8)<br>min-max: [203.0-422.0]	mean: 655.0<br>median: 544.0 (IQR: 263.5)<br>min-max: [427.0-1600.0]
**Sex, n (%)**	Female: 74 (42.3%)<br>Male: 101 (57.7%)	Female: 26 (44.1%)<br>Male: 33 (55.9%)	Female: 27 (46.6%)<br>Male: 31 (53.4%)	Female: 21 (36.2%)<br>Male: 37 (63.8%)
**Age at diagnosis (years)**	mean: 67.7<br>median: 332.0 (IQR: 349.0)<br>min-max: [25.5-88.9]	mean: 71.6<br>median: 72.2 (IQR: 11.0)<br>min-max: [25.5-88.9]	mean: 67.1<br>median: 67.7 (IQR: 14.6)<br>min-max: [46.9-85.3]	mean: 64.7<br>median: 64.9 (IQR: 13.2)<br>min-max: [42.3-83.7]
**Resection type, n (%)**	Biopsy: 69 (39.4%)<br>Subtotal: 62 (35.4%)<br>Gross-total: 44 (25.1%)	Biopsy: 37 (62.7%)<br>Subtotal: 18 (30.5%)<br>Gross-total: 4 (6.8%)	Biopsy: 21 (36.2%)<br>Subtotal: 19 (32.8%)<br>Gross-total: 18 (31.0%)	Biopsy: 11 (19.0%)<br>Subtotal: 25 (43.1%)<br>Gross-total: 22 (37.9%)
**WHO performance status, n (%)**	0: 27 (15.4%)<br>1: 95 (54.3%)<br>2: 45 (25.7%)<br>3: 8 (4.6%)<br>4: 0 (0.0%)<br>5: 0 (0.0%)	0: 3 (5.1%)<br>1: 25 (42.4%)<br>2: 25 (42.4%)<br>3: 6 (10.2%)<br>4: 0 (0.0%)<br>5: 0 (0.0%)	0: 12 (20.7%)<br>1: 32 (55.2%)<br>2: 13 (22.4%)<br>3: 1 (1.7%)<br>4: 0 (0.0%)<br>5: 0 (0.0%)	0: 12 (20.7%)<br>1: 38 (65.5%)<br>2: 7 (12.1%)<br>3: 1 (1.7%)<br>4: 0 (0.0%)<br>5: 0 (0.0%)

Supplementary table 2: Description of the patient cohort for four-class survival outcome prediction.

Category	All Patients (n=175)	Class 1 (n=44) OS ≤ 147 days	Class 2 (n=45) 147 days < OS ≤ 332 days	Class 3 (n=42) 332 days < OS ≤ 492 days	Class 4 (n=44) OS > 492 days
**Survival (days)**	mean: 363.4<br>median: 332.0 (IQR: 349.0)<br>min-max: [42.0-1600.0]	mean: 93.9<br>median: 102.5 (IQR: 57.0)<br>min-max: [42.0-138.0]	mean: 242.9<br>median: 238.0 (IQR: 124.0)<br>min-max: [149-332.0]	mean: 394.2<br>median: 389.0 (IQR: 80.8)<br>min-max: [336.0-492.0]	mean: 707.0<br>median: 653.0 (IQR: 329.8)<br>min-max: [493.0-1600.0]
**Sex, n (%)**	Female: 74 (42.3%)<br>Male: 101 (57.7%)	Female: 20 (45.5%)<br>Male: 24 (54.5%)	Female: 21 (46.7%)<br>Male: 24 (53.3%)	Female: 18 (42.9%)<br>Male: 24 (53.3%)	Female: 15 (34.1%)<br>Male: 29 (64.4%)
**Age at diagnosis (years)**	mean: 67.7<br>median: 332.0 (IQR: 349.0)<br>min-max: [25.5-88.9]	mean: 72.7<br>median: 73.2 (IQR: 9.5)<br>min-max: [31.7-88.9]	mean: 68.3<br>median: 69.3 (IQR: 12.8)<br>min-max: [25.5-85.3]	mean: 64.5<br>median: 67.1 (IQR: 14.2)<br>min-max: [42.3-83.7]	mean: 64.5<br>median: 65.3 (IQR: 13.5)<br>min-max: [46.3-80.9]
**Resection type, n (%)**	Biopsy: 69 (39.4%)<br>Subtotal: 62 (35.4%)<br>Gross-total: 44 (25.1%)	Biopsy: 28 (63.6%)<br>Subtotal: 14 (31.8%)<br>Gross-total: 2 (4.5%)	Biopsy: 23 (51.1%)<br>Subtotal: 15 (33.3%)<br>Gross-total: 7 (15.6%)	Biopsy: 11 (24.4%)<br>Subtotal: 13 (31.0%)<br>Gross-total: 18 (40.0%)	Biopsy: 7 (15.6%)<br>Subtotal: 20 (45.5%)<br>Gross-total: 17 (37.8%)
**WHO performance status, n (%)**	0: 27 (15.4%)<br>1: 95 (54.3%)<br>2: 45 (25.7%)<br>3: 8 (4.6%)<br>4: 0 (0.0%)<br>5: 0 (0.0%)	0: 3 (6.8%)<br>1: 17 (38.6%)<br>2: 19 (43.2%)<br>3: 5 (11.4%)<br>4: 0 (0.0%)<br>5: 0 (0.0%)	0: 4 (8.9%)<br>1: 25 (55.6%)<br>2: 14 (31.1%)<br>3: 2 (4.4%)<br>4: 0 (0.0%)<br>5: 0 (0.0%)	0: 9 (20.0%)<br>1: 27 (64.3%)<br>2: 5 (11.9%)<br>3: 1 (2.4%)<br>4: 0 (0.0%)<br>5: 0 (0.0%)	0: 11 (24.4%)<br>1: 26 (59.1%)<br>2: 7 (15.9%)<br>3: 0 (0.0%)<br>4: 0 (0.0%)<br>5: 0 (0.0%)

Supplementary table 3:
Slice-level performance of non-AI and AI models for the prediction of survival outcome. Values are point estimates with 95% confidence intervals. CIs were obtained by patient-level bootstrapping (2,000 resamples). For multiclass tasks, metrics were computed per class (one-vs-rest) and macro-averaged across classes.

Model	Area Under the Curve (AUC)	Specificity (%)	Sensitivity (%)	Accuracy (%)	Precision (%)	F1 (%)
Two-class model: MRI only	0.890 (0.779–0.976)	90.74 (79.53–99.15)	73.10 (53.80–90.48)	81.68 (70.27–92.19)	89.29 (75.00–99.15	80.39 (65.08–91.67)
Three-class model: MRI only	0.936 (0.872–0.983)	90.88 (85.25–95.74)	81.77 (71.07–91.33)	81.77 (70.66–91.45)	82.29 (71.62–91.65)	81.81 (70.30–91.15)
Four-class model: MRI only	0.903 (0.816-0.967)	90.47 (85.75–94.93)	71.67 (57.29–85.28)	71.05 (56.43–84.80)	75.51 (62.91–87.02)	71.30 (55.20–84.55)
